# The change in liver stiffness, controlled attenuation parameter and fibrosis-4 index for chronic hepatitis C patients with direct-acting antivirals

**DOI:** 10.1371/journal.pone.0214323

**Published:** 2019-04-02

**Authors:** Yu-Chi Lee, Tsung-Hui Hu, Chao-Hung Hung, Sheng-Nan Lu, Chien-Hung Chen, Jing-Houng Wang

**Affiliations:** 1 Division of Hepato-Gastroenterology, Department of Medicine, Kaohsiung Chang Gung Memorial Hospital and Chang Gung University, Kaohsiung City, Taiwan; 2 Division of Hepato-Gastroenterology, Department of Medicine, Chia-Yi Chang Gung Memorial Hospital, Chia-Yi City, Taiwan; National Taiwan University Hospital, TAIWAN

## Abstract

**Background and aim:**

Transient elastography and fibrosis-4 index (FIB-4) have been proposed to access hepatic fibrosis and steatosis for patients with chronic liver disease. This study was to determine the changes of liver stiffness (LS), controlled attenuation parameter (CAP) value and FIB-4 and their associated factors for chronic hepatitis C (CHC) patients who underwent direct-acting antivirals (DAAs).

**Patients and methods:**

Consecutive patients with CHC in advanced fibrosis or compensated cirrhosis undergoing paritaprevir/ritonavir/ombitasvir plus dasabuvir therapy and with LS and CAP before and 12 weeks after treatment were enrolled. The demographics, clinical characteristics and treatment outcomes were reviewed. The changes of LS, FIB-4, CAP and their associated factors were analyzed.

**Results:**

A total of 213 patients (mean age: 63.7 years) with complete recommended treatment were enrolled. All patients achieved sustained virological response at 12 weeks (SVR12) of follow-up. The mean values of LS, CAP and FIB-4 index before treatment were 18.5kPa, 283dB/m and 5.05 respectively. While there was no significant change in CAP, LS and FIB-4 decreased significantly at the time of SVR12 (p<0.001). Compared with follow-up period, LS and FIB-4 decreased rapidly during DDAs. Multivariate analysis showed that higher baseline LS and FIB-4 were associated with greater reductions at the time of SVR12.

**Conclusion:**

For CHC patients in advanced fibrosis or compensated cirrhosis, DAAs improved LS and FIB-4 index at SVR12. Higher baseline LS and FIB-4 contributed to greater reductions. However, there was no significant change in CAP value.

## Introduction

Chronic hepatitis C virus (HCV) infection affects over 71 million people worldwide and leads to significant morbidity and mortality through its predisposition to liver fibrosis, cirrhosis, and liver cancer [1]. Each year, hepatitis C causes approximately 399,000 deaths worldwide, mostly from cirrhosis and hepatocellular carcinoma (HCC) [2]. Newer combination of direct-acting antivirals (DAAs) offers cure rates exceeding 90% [3]. However, achieving sustained virologic response (SVR) does not immediately reverse HCV-related liver fibrosis or cirrhosis. The DAAs regimen, paritaprevir/ritonavir/ombitasvir plus dasabuvir (PrOD) regimen, had been used for the treatment of genotype 1 chronic hepatitis C virus infection [4]. However, there is little or no information on whether the PrOD affects liver fibrosis.

The severity of liver fibrosis is associated with the prognosis of liver disease. Liver biopsy is still considered the gold standard for evaluation of liver fibrosis although it is an invasive procedure. Some non-invasive procedures, including liver stiffness (LS) and fibrosis-4 (FIB-4) index measurement, have been recently reported for longitudinal evaluation of liver fibrosis [5]. Currently, transient elastography (TE) is considered the non-invasive standard for the measurement of LS and widely used for the assessment of liver fibrosis with high accuracy in diagnosing severe fibrosis and cirrhosis [5–7]. With the enhancement of TE machine including a new software, it is now possible to calculate the so called controlled attenuation parameter (CAP), which is a reliable noninvasive quantitative marker of hepatic steatosis [8, 9].There were reports showing the changes of LS and CAP values for HCV patients underwent DAAs therapy [10, 11]. The purpose of present study was to determine the changes of LS, CAP, FIB-4 index and their associated factors for patients with HCV-related and compensated liver disease underwent PrOD therapy.

## Patients and methods

### Patients

From January 2017 to August 2017, there were 337 patients with chronic HCV genotype 1 infection and underwent PrOD therapy in this hospital. We enrolled into this study those patients with the diagnosis of advanced fibrosis and/or compensated cirrhosis, defined by FIB-4 index ≥3.25 and/or LS≥9.5kPa, with recommended treatment course and available HCV-RNA results 12 weeks after end of therapy, and with reliable LS results before and 12 weeks after end of treatment (EOT). Sustained virological response (SVR) was defined as undetectable HCV-RNA 12 weeks after EOT. FIB-4 index is obtained based on four factors including age, aspartate aminotransferase, alanine aminotransferase and platelet count. The demographics and clinical characteristics of enrolled patients were reviewed and recorded. The study was approved by the Institution Review Board and Ethics Committee of Chang Gung Memorial Hospital, Kaohsiung, Taiwan (IRB201801403B0). The Ethics Committee waived the requirement for informed consent accordance with the relevant guidelines and regulations. There was no funders in study design, data collection and analysis, decision to publish, or preparation of the manuscript. The authors have declared that no competing interests exist.

### Liver stiffness and controlled attenuation parameter

LS and CAP were measured using the TE (FibroScan-502, Echosens, Paris, France) with M- or XL-probe by one technician with more than 5 years of experience in performing TE. Patients were placed in a supine position with the right hand at the most abducted position for right lobe liver scanning. When at least 10 valid measurements were obtained with valid measurements at ≥60% and interquartile range of <30%, such measurements were considered valid and the median value of these measurements was used for analysis. The CAP represents ultrasonic attenuation of the liver at 3.5 MHz using signals acquired by the probe. CAP is measured only on validated measurements based the same criteria used for measurement of LS and on the same signals.

### Statistical analysis

The result of LS and CAP was expressed as median value with interquartile range (IQR) in kilopascals (kPa) and dB/m. Quantitative variables were expressed with mean ± SD or median with a range. Qualitative variables were expressed as absolute and relative frequencies. We performed multivariable linear regression analysis to control for confounding by variables. Differences in nonparametric variables between groups were compared by the pair t test. Continuous variables were analyzed using linear regression, while categorical variables were analyzed using Chi-square test. All P values of <0.05 by the two-tailed test were considered significant. All statistical analysis was carried out using SPSS software (SPSS Inc., Chicago, IL)

## Results

### Patient characteristics

A total of 213 patients (male/female: 97/116, mean age:63.7 years) were enrolled. There were 21 patients with genotype 1a and 192 genotype 1b underwent 24 weeks and 12 weeks PrOD therapy respectively. Fatty liver was diagnosed by sonography in 58 (27.2%) patients. There was 11 (5.1%) patients with the presence of hepatitis B virus surface antigen. Eighteen (8.5%) patients were with hepatocellular carcinoma post curative treatments. For all patients, there were no histories of congestive heart failure, human immunodeficiency virus infection, autoimmune hepatitis or significant alcohol consumptions at the reviews of medical records. The mean values of LS, CAP and FIB-4 index before treatment were 18.5kPa, 283dB/m and 5.05 respectively **(**Table 1). All patients achieved SVR at 12 weeks (SVR12) after EOT.

**Table 1:** Demographics and baseline clinical characteristics.

Characteristics	N = 213
Age (years, mean±SD,)	63.7±9.6
Sex, n (%)	
Male	97 (45.5%)
Female	116 (54.5%)
Body mass index (kg/m^2^, mean±SD)	25.1±3.9
Genotype, n (%)	
1a	21 (9.9%)
1b	192 (90.1%)
Cirrhosis, n (%)	116 (54.5%)
Hepatocellular carcinoma, n (%)	18 (8.5%)
Fatty liver by sonography	58(27.2%)
HBsAg presence, n (%)	11(5.1%)
AST (U/L, mean±SD)	81.3±55.8
ALT (U/L, mean±SD)	131.1±267.4
Albumin (mg/dl, mean±SD)	4.29±0.34
Baseline HCV RNA(log IU/ml, mean±SD)	5.99±0.75
Platelet (10^3^/mm^3^, mean±SD)	140.2±67.4
Fibrosis-4 index ^†^ (mean±SD)	5.05±6.96
Prothrombin time (INR, mean±SD)	1.16±0.91
Total Bilirubin (mg/dl, mean±SD)	1.02±0.39
Total cholesterol ^#^ (mg/dl mean±SD)	167.7±31.5
Triglyceride ^#^ (mg/dl, mean±SD)	99.3±55.6
Alpha-Fetoprotein (ng/ml, mean±SD)	17.8±37.5
Baseline liver stiffness (kPa, mean±SD)	18.5±11.6
Baseline CAP (dB/m, mean±SD)	228.0±35.6

HBsAg, hepatitis B virus surface antigen; AST, aspartate aminotransferase; ALT, alanine aminotransferase; CAP, controlled attenuation parameter

^†^: Fibrosis-4 index = [(Age * AST) / (PLT* (ALT)^1/2^)]

^#^: n = 155

### Changes in LS, CAP and FIB-4 index

**Fig 1:**
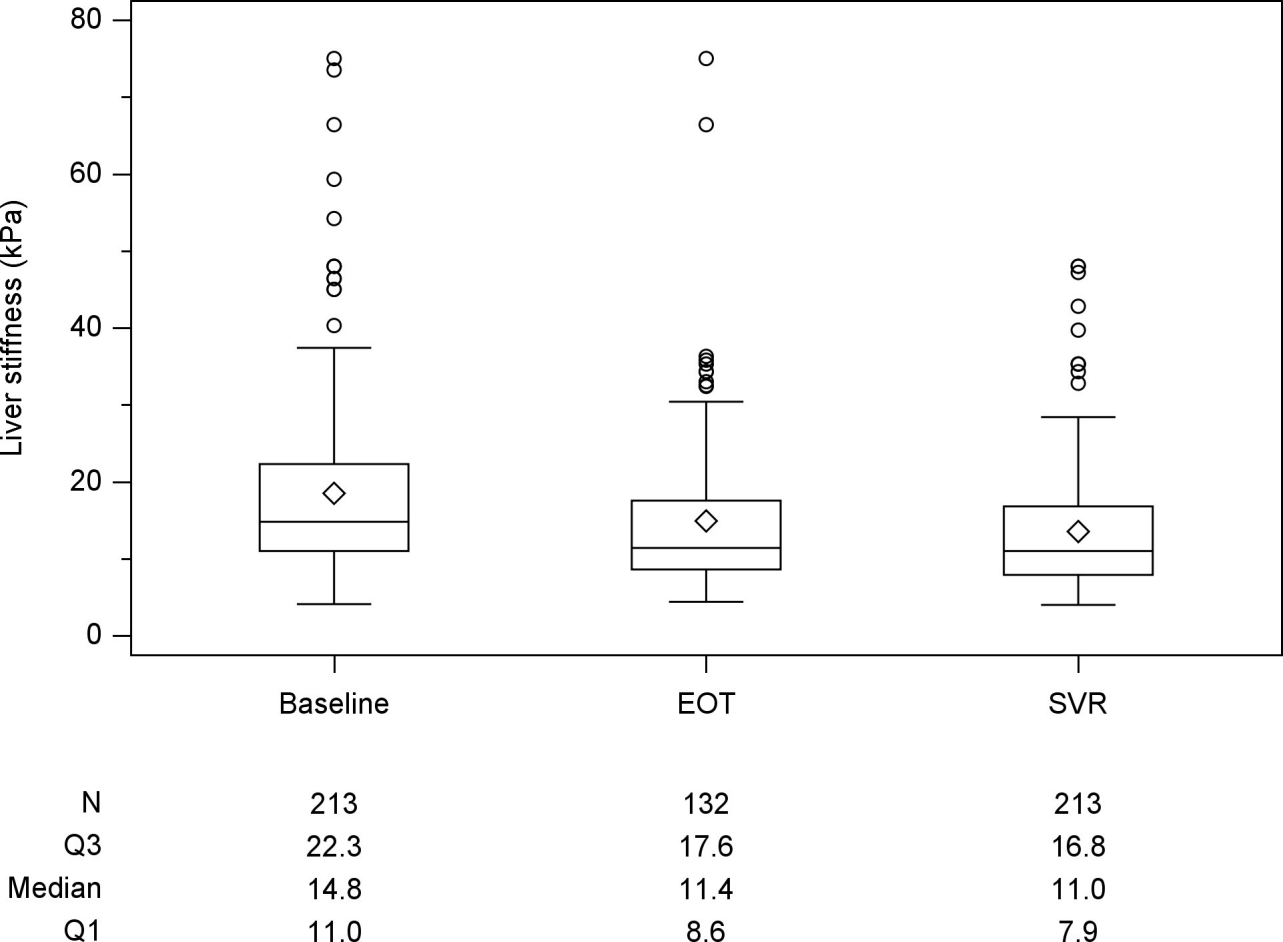
Box plot of liver stiffness (LS) at the time of baseline, end of treatment (EOT)and sustained of virological response (SVR) 12 weeks after EOT. Compared with baseline LS, there is significant reduction at the time of SVR12 (p<0.001).

**Fig 2:**
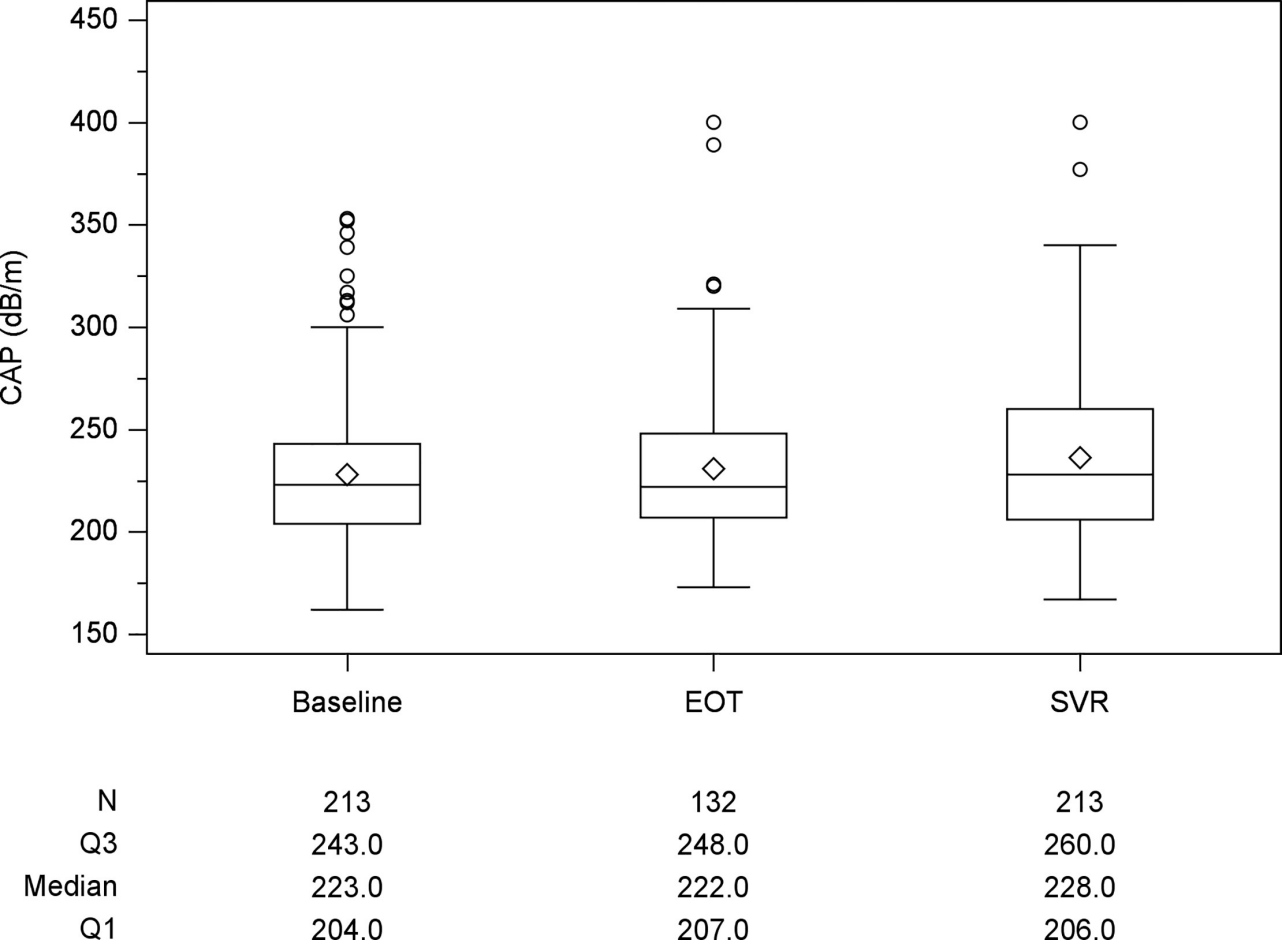
Box plot of controlled attenuation parameter (CAP) at the time of baseline, end of treatment (EOT) and sustained of virological response (SVR) 12 weeks after EOT. Compared with baseline CAP, there is no significant change at the time of SVR12 (p = 0.227).

**Fig 3:**
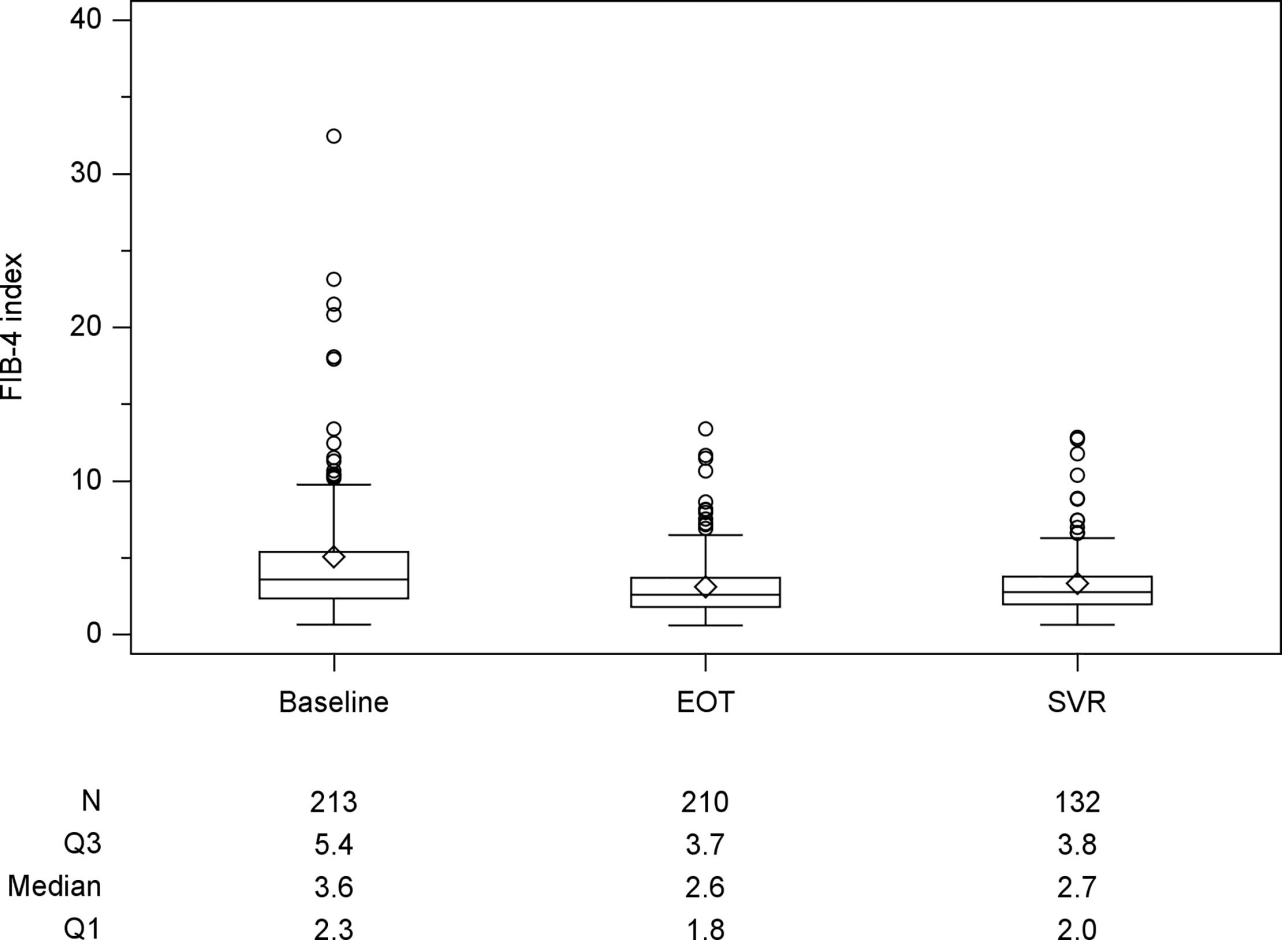
Box plot of fibrosis-4 index (FIB-4) at the time of baseline, end of treatment (EOT) and sustained of virological response (SVR) 12 weeks after EOT. Compared with baseline FIB-4, there is significant reduction at the time of SVR12 (p<0.001).

The median LS values for all patients at baseline and SVR12 were 14.8kPa and 11.0kPa, respectively. Compared to baseline value, there were 185 (86.9%) patients with decline of LS and 28 (13.1%) with increase at SVR12. There were 132 patients received LS measurement at EOT with a median LS value of 11.4kPa (Fig 1). There was a significant decline in LS values from baseline to EOT (P < 0.001) and baseline to SVR12 (p < 0.001). The median CAP values were 223 and 228 dB/m at baseline and SVR12 (Fig 2). There were 132 patients with CAP measurement at EOT with a median CAP values 222 dB/m. Although CAP level increased from baseline to SVR12, there was no significant change statistically. Stratified baseline CAP level by 236 dB/m, there was significant increase at SVR12 for those patients with baseline level <236 dB/m (baseline vs SVR12: 210.0 vs 220.0, p<0.0001). However, there was no significant change for those patients with baseline level ≥236 dB/m (baseline vs SVR12: 261 vs 256, p = 0.198). The median FIB-4 index values at baseline and SVR12 were 3.6 and 2.7 (Fig 3). There was a significant decline in FIB-4 index values from baseline to EOT (p<0.001) and baseline to SVR12 (p<0.001). Compared with those during follow-up period, the magnitudes in changes of LS and FIB-4 were greater during DAAs therapy (Table 2). There was a rapid decline during DAAs therapy. For those patients with baseline platelet count <150x10^3^/mm^3^, there was significant increase in platelet at SVR12 (109x10^3^/mm^3^ vs 117 x10^3^/mm^3^, p<0.0001).

**Table 2:** The magnitude of change in liver stiffness, controlled attenuation parameter (CAP) and fibrosis-4 (FIB-4) index during treatment (baseline-end of treatment, EOT) and follow-up (EOT- sustained virological response at 12 weeks, SVR12) periods.

	Baseline-EOT (%)	EOT-SVR12 (%)	
Variables (n)	Mean±SD	Mean±SD	p-value^a^
Liver stiffness (132)	-17.57±25.54	-7.03±23.48	0.0019
CAP (132)	1.90±16.03	3.83±15.66	0.1746
FIB-4 (129)	-23.81±31.59	7.68±25.76	<0.0001

^a^: Wilcoxon signed-rank test

### The factors associated with changes in LS and FIB-4 index

Uni-variate analysis showed baseline body mass index (p = 0.03), platelet count (p = 0.05), triglyceride (p = 0.004) and baseline LS value (p<0.001) were the associated with LS changes. We selected these factors for multivariate analysis and showed baseline LS was the associated factor for the magnitude in changes of LS value (p<0.001). Higher baseline LS value contributed to greater reduction at SVR 12 (Table 3). While higher baseline FIB-4 index and platelet count contributed the greater reduction at SVR12, old age was associated with an increase in FIB-4 index (Table 4).

**Table 3:** Uni- and multi-variate analysis of factors associated with the magnitude of changes in liver stiffness (LS).

Baseline variables	Univariate			Multivariate		
	β	SE	p-value	β	SE	p-value
Age (years)	-0.068	0.064	0.2889			
Sex (Male: Female)	1.250	1.140	0.2745			
HCC (Yes: No)	-2.887	2.036	0.1583			
BMI (kg/m^2^)	-0.331	0.154	0.0339			
Prothrombin time (INR)	0.119	0.532	0.8238			
AST (U/L)	-0.010	0.010	0.3160			
ALT (U/L)	0.0001	0.0018	0.9683			
Total bilirubin (mg/dl)	0.234	1.558	0.8809			
Platelet (1000/mm^3^)	0.015	0.008	0.0508			
Alpha-fetoprotein (ng/mL)	-0.001	0.014	0.9287			
HCVRNA (IU/mL, log)	-0.092	0.824	0.9117			
FIB-4 index	-0.105	0.073	0.1542			
Baseline-LS (kPa)	-0.415	0.033	<0.0001	-0.415	0.033	<0.0001
Baseline-CAP (dB/m)	-0.038	0.016	0.0181			
Triglyceride (mg/dl)	-0.029	0.010	0.0041			
Total cholesterol (mg/dl)	0.010	0.018	0.5804			

HCC: hepatocellular carcinoma; BMI: body mass index; AST: aspartate aminotransferase; ALT: alanine aminotransferase; FIB-4: fibrosis-4; CAP: controlled attenuation parameter

**Table 4:** Uni- and multi-variate analysis of factors associated with the magnitude of changes in fibrosis-4 index (FIB-4).

Baseline variables	Univariate			Multivariate		
	β	SE	p-value	β	SE	p-value
Age (years)	0.167	0.096	0.0856	0.062	0.015	<0.0001
Sex (Male: Female)	-1.464	1.724	0.3978			
HCC (Yes: No)	0.273	3.187	0.9318			
BMI (kg/m^2^)	0.085	0.237	0.7208			
Prothrombin time (INR)	0.125	0.654	0.8485			
AST (U/L)	-0.091	0.010	<0.0001			
ALT (U/L)	0.0020	0.0023	0.3743			
Total bilirubin (mg/dl)	0.958	2.457	0.6975			
Platelet (1000/mm^3^)	0.037	0.016	0.0204	-0.022	0.003	<0.0001
Alpha-fetoprotein (ng/mL)	-0.005	0.029	0.8622			
HCVRNA (IU/mL, log)	-1.259	1.273	0.3248			
FIB-4 index	-0.906	0.020	<0.0001	-0.947	0.015	<0.0001
Baseline-LS (kPa)	-0.024	0.070	0.7267			
Baseline-CAP (dB/m)	-0.000	0.025	0.9927			
Triglyceride (mg/dl)	0.009	0.014	0.5190			
Total cholesterol (mg/dl)	-0.003	0.026	0.9043			

HCC, hepatocellular carcinoma; BMI, body mass index; AST, aspartate aminotransferase; ALT, alanine aminotransferase; FIB-4, fibrosis-4; CAP, controlled attenuation parameter

## Discussion

The major goals for CHC treatment are achieving high rates of SVR and improving HCV-induced liver fibrosis. DAAs does not have an anti-fibrotic effect but mainly concerned with viral eradication[12, 13]. Liver stiffness and CAP by TE has been proposed to access hepatic fibrosis and steatosis for patients with chronic liver disease. Liver stiffness decreases significantly after SVR with antiviral treatment [10–12, 14–16]. Our study demonstrated that chronic HCV patients with severe fibrosis or compensated cirrhosis achieved 100% SVR 12 rate after recommended PrOD treatment course. And there were significant improvements of LS and FIB-4 index. However, there was no significant change between baseline and SVR12 in CAP value. Compared with follow-up period, LS and FIB-4 index decreased rapidly during treatment. Higher baseline LS and FIB-4 index values were associated with greater reductions in LS and FIB-4 index values at the time of SVR12.

Recent studies have shown significant LS decrease between baseline and SVR24 for chronic HCV patients with genotype 1b or 2 underwent different DAA therapy [10–12, 14–16]. The magnitude of decline for patients underwent DAA therapy, which was higher than that with interferon-based therapy [17]. PrOD therapy has been demonstrated to improve the histopathological features regardless of the treatment efficacy [12, 14]. Our study further demonstrated there were continuous and significant decrease in LS and FIB-4 index from baseline to SVR12 for patients with PrOD therapy. LS decreased in SVR patients during a short period of time [10, 11, 14]. DAA management for chronic hepatitis C showed the LS changes decreased at EOT after ledipasvir/sofosbuvir treatment (median baseline LS:8.3 kPa, EOT:7.4 kPa) [10]. Our study showed that there was greater LS decline at EOT after PrOD treatment than the decline at follow-up period. However, LS is influenced not only by the degree of liver fibrosis, but also by inflammatory activity [18, 19]. FIB-4 index has also been demonstrated significant decrease in CHC patients after Sofosbuvir-based treatment and asunaprevir dual therapy (mean decline 12.2%) [11, 16]. In our study mean FIB-4 index decline was 23.8% at baseline and EOT after PrOD treatment. This result was compatible with the inflammatory activity improved after DAA treatment in the short period. The greater declines of LS and FIB-4 index in our study might be explained by the different DAAs treatment. Whether PrOD treatment for chronic hepatitis C patient yielded a greater viral suppression resulted in improvement of inflammation and fibrogenesis was unknown. However, it remained to be examined whether this indicates a true regression of fibrosis or merely resolution of chronic liver inflammation with subsequent improvement and laboratory parameters [15, 20].

Many investigators have reported that interferon-based treatment is effective in reducing serum alanine amino-transferase levels, eradicating HCV RNA and reducing liver fibrosis in patients with chronic HCV infection. Compared with LS in patients treated with pegylated interferon plus ribavirin [21], our study showed greater LS decline from baseline to SVR. This result demonstrated that there might be a greater decrease and proportion of patients with improvement of LS after DAA treatment than those after interferon-based treatment.

The prevalence of liver steatosis is strongly associated with HCV genotype [21]. Liver steatosis decreased significantly from baseline to SVR24 for chronic HCV patients with ledipasvir/sofosbuvir or ombitasvir/paritaprevir/ritonavir treatment [14]. However, there were no significant CAP changes overall and between various timepoints (EOT, SVR24, SVR48) in patients treated with ledipasvir/sofosbuvir [10]. Our study showed no significant change in CAP value between baseline and SVR12. In subgroup analysis, CAP level showed significant increase from baseline to SVR 12 for those patients with CAP<236 dB/m. The issue of CAP changes in HCV genotype 1 patients after DAA treatment is still controversial [10, 11, 14]. Therefore, further studies are required to investigate the relation between changes in CAP following DAA treatment.

This study has certain limitations. All subjects were patients with advanced fibrosis or compensated cirrhosis, enrolled from single medical center and infected with HCV genotype 1. Accordingly, generalization of our results can only be made for HCV patient with advanced fibrosis or compensated cirrhosis underwent PrOD treatment. There were 11 patients co-infected with hepatitis B virus and 58 patients with fatty liver by sonography, which might have impact on baseline liver stiffness and its change. In this study, all patients were followed until SVR12, which clinical outcomes of LS improvement were not evaluated in this short-term period of follow-up. Therefore, long-term follow up is necessary to clarify whether the improvements of LS and FIB-4 index during DAA therapy reflects true regression of fibrosis and the durability of LS and FIB-4 index decreasing after SVR12.

In summary, our study demonstrated the decline in LS and FIB-4 index at end of PrOD treatment and 12 weeks follow-up. Compared with follow-up period, LS and FIB-4 index decreased more rapidly from baseline to EOT. Higher initial LS value showed greater LS value reduction at SVR 12. The higher baseline platelet count and FIB-4 index value showed greater reduction in FIB-4 index at the time of SVR12. Our study showed no significant change in CAP, however, there was a tendency for increase in CAP value. Further longitudinal study is needed to evaluate the clinical significance of this short-term change in LS and CAP.

## Supporting information

S1 Dataset This file provides the data of yearly liver stiffness changes & associated factors p-values. (XLS)
